# The frequency of acid-base disorders on admission to the intensive care and its association with in-hospital outcome, Cape Town, South Africa: a retrospective cohort study

**DOI:** 10.11604/pamj.2022.42.130.32570

**Published:** 2022-06-16

**Authors:** Nokwanda Sithole, Usha Lalla, Mogamat Razeen Davids, Mogamat-Yazied Chothia

**Affiliations:** 1 Division of Nephrology, Department of Medicine, Faculty of Medicine and Health Sciences, Stellenbosch University, Cape Town, South Africa,; 2 Division of Pulmonology, Department of Medicine, Faculty of Medicine and Health Sciences, Stellenbosch University, Cape Town, South Africa

**Keywords:** Acid-base disorders, anion gap, mortality, South Africa

## Abstract

**Introduction:**

acid-base disorders are very common in critically ill patients and contribute significantly to morbidity and mortality. The aim of this study was to identify the types of acid-base disorders at the time of admission to the intensive care unit (ICU) and its associated ICU and in-hospital mortality.

**Methods:**

we conducted a retrospective cohort study of all adult patients that were admitted to the ICU and had an arterial blood gas sample at the time of admission from 1^st^ January 2019 to 31 December 2019. Using the traditional approach, acid-base disorders were categorised into six disorders. Variables predicting in-hospital death were identified using logistic regression.

**Results:**

a total of 375 patients were included. The median age for the entire cohort was 39 (IQR 30–52) years and 48.3% (n=181) were female. Mixed acid-base disorders were the most common at 48.8% (n=183), followed by no disorder at 24.8% (n=93), metabolic acidosis at 9.3% (n=35), metabolic alkalosis at 6.7% (n=25), respiratory acidosis 6.1% (n=23) and respiratory alkalosis at 4.3% (n=16). A total of 94 (25.0%) patients died. There were no differences in ICU (p = 0.35) or in-hospital death (p = 0.32) by acid-base disorder. Male sex (aOR: 5.8, 95% CI 1.55-21.42; p < 0.01), APACHE II score (aOR: 1.17, 95% CI 1.06-1.30; p < 0.01) and the corrected anion gap (aOR: 1.14, 95% CI 1.02-1.27; p = 0.02) were identified as predictors of in-hospital death using multivariable logistic regression.

**Conclusion:**

there was no association between acid-base disorders at the time of ICU admission and ICU or in-hospital death. Therefore, in our setting, acid-base disorders at the time of ICU admission should not be used to predict the outcome of patients requiring intensive care.

## Introduction

Acid-base disorders are very common in critically ill patients [1]. There is no single acid-base disorder that is consistently the most common in the intensive care unit (ICU) [2, 3]. One study reported that as many as 70% of patients had a mixed acid-base disorder at the time of ICU admission with metabolic acidosis-respiratory alkalosis being the most common [4]. Metabolic acidosis is categorised according to the presence or absence of new unmeasured anions, inferred by calculating the anion gap (AG). An elevated AG metabolic acidosis is common in critically ill patients. Its presence reflects the addition of acids such as endogenously produced lactic acid and ketoacids, an inability to excrete organic acid anions during kidney failure or the exogenous intake of toxins such as ethylene glycol or intravenous drugs containing propylene glycol [5, 6]. It is associated with an increased severity of illness that is independent of concomitant electrolyte abnormalities [6, 7]. Furthermore, an increased AG was associated with the highest mortality especially when it was greater than 30 mmol/L, even after adjusting for aetiology [8].

Metabolic alkalosis is often considered the most common acid-base disorder encountered in the ICU [9-11]. It is associated with a poor prognosis, with reported mortality rates as high as 80% when blood pH is greater than 7.65 [11]. The prognosis is compounded when metabolic alkalosis is associated with respiratory alkalosis [12]. The poor prognosis is the result of the severity of the underlying disease [11]; however, severe metabolic alkalosis by itself may have detrimental consequences in acutely ill patients, including hypoventilation and an increased risk of cardiac arrythmias [13]. Common causes in critically ill patients include diuretics, vomiting and nasogastric tube drainage, steroid use and post-hypercapnic alkalosis in mechanically-ventilated patients with chronic obstructive pulmonary disease [14].

In an analysis of 8 607 consecutive arterial blood gas samples, respiratory alkalosis was observed in 45%. On the other hand, respiratory acidosis was seen in only 13%, suggesting that hyperventilation is considerably more common than hypoventilation in critically ill patients [13]. Extreme hypocapnia in the critically ill was found to have serious prognostic implications and was indicative of the severity of the underlying disease [13]. Alternatively, compared to compensated or normocarbic patients, mechanically-ventilated patients with hypercapnic acidosis had increased mortality during the first 24-hours of intensive care [7].

Differences in outcomes exist between metabolic acidosis and respiratory acidosis with similar pH ranges. This observation suggests that the underlying disorder may be more important than the absolute degree of acidaemia [15]. However, some causes of metabolic acidosis seem to have worse outcomes than others. In a study that compared the mortality between lactic acidosis and other causes of metabolic acidosis, a higher in-hospital mortality was found in patients with lactic acidosis [5]. This finding is not surprising since lactic acidosis has been shown to be associated with high mortality in critically ill patients [16]. Using the traditional approach, the aim of this study was to determine the frequency and types of acid-base disorders at the time of admission to the ICU and to assess whether acid-base disorders were associated ICU and in-hospital mortality.

## Methods

**Study design and setting:** we conducted a retrospective cohort study from 1^st^ January 2019 to 31 December 2019 of patients admitted to the medical ICU at Tygerberg Hospital in Cape Town, South Africa. Tygerberg Hospital is a 1340 bed facility that offers tertiary level healthcare to patients dependent on the public sector.

**Data collection:** data were extracted from patient charts and included demographic data, comorbidities and primary diagnosis, medical or surgical discipline, and clnical data including Acute Physiology and Chronic Health Evaluation II (APACHE II) score, blood pressure, acute kidney injury and dialysis requirements. As a result of resource constraints only patients that required or were currently receiving mechanical ventilation in the emergency department were evaluated by the ICU team for admission. Routine arterial blood gas samples were performed on all patients via an arterial line at the time of admission to the ICU. These were used to screen for an acid-base disorder. All samples were processed using the GEM Premier 3500 or 4000 systems (Ilex Medical, South Africa). The equipment undergoes internal calibration whenever a new reagent pack is inserted. All data were collected into an Excel spreadsheet.

**Study population:** patients included in the study were nearly exclusively mechanically ventilated adult patients (age ≥ 18 years). Patients were excluded if they were readmitted to the ICU. We estimated a sample size of 208 patients with a confidence interval of 95%, a margin of error of 5%, 50% of the population having an acid-base disorder and a total population of 448 patients admitted to the intensive care during the study period.

### Definitions

Using the traditional approach [16], patients were grouped into six acid-base disorders, with their secondary “adaptive” responses, as follows [17]: metabolic acidosis: pH <7.38; [HCO_3_] <22 mmol/L; adaptive respiratory response (Winter´s formula): PaCO_2_ (kPa) = {1.5 x [HCO_3_] + 8 ± 2} x 0.133; mixed respiratory acidosis or alkalosis was present when the calculated PaCO_2_ was higher or lower than expected. **Metabolic alkalosis:** pH >7.42; [HCO_3_] >26 mmol/L; adaptive respiratory response: PaCO_2_ (kPa) = {0.7 x ([HCO_3_] - 24) + 40 ± 2} x 0.133; mixed respiratory acidosis or alkalosis was present when the calculated PaCO_2_ was higher or lower than expected. **Respiratory acidosis:** pH <7.38; PaCO_2_ >5.6 kPa; adaptive metabolic response: **acute**- [HCO_3_] increases by 1 mmol/L for every PaCO_2_ increase of 1.3 kPa above 5.3 kPa; **chronic**- [HCO_3_] increased by 4-5 mmol/L for every PaCO_2_ increase of 1.3 kPa above 5.3 kPa; mixed metabolic acidosis or alkalosis was present when the calculated [HCO_3_] is higher or lower than expected. **Respiratory alkalosis:** pH >7.42; PaCO_2_ <4.5 kPa; adaptive metabolic response: **acute**- [HCO_3_] decreases by 2 mmol/L for every PaCO_2_ decrease of 1.3 kPa below 5.3 kPa; **chronic**- [HCO_3_] decreased by 4-5 mmol/L for every PaCO_2_ decrease of 1.3 kPa below 5.3 kPa; mixed metabolic acidosis or alkalosis may be present when the calculated [HCO_3_] was higher or lower than expected. Normal serum lactate was defined as a value less than 2 mmol/L. The anion gap (AG) was calculated using the following formula: [Na^+^+ K^+^] - [Cl^-^- HCO_3_], and the reference range of 16 ± 2 mmol/L was used. The reference range for serum albumin was 35 to 52 g/L. Since serum albumin is the major anion that contributes to the AG, the latter was adjusted by a correction factor of 2.5 mmol/L for every change of 10 g/L in the serum albumin concentration.

**Ethical considerations:** as this was a retrospective study, permission was granted for a waiver of informed consent. This study was approved by the Health Research Ethics Committee (HREC) of Stellenbosch University (HREC reference number: S20/04/089, Project identification: 15057). This study was conducted in accordance with the principles of the Declaration of Helsinki.

**Statistical analysis:** descriptive numerical data with a normal distribution were summarised using mean and standard deviation (SD) while the median and interquartile range (IQR) was used for data that were not normally distributed. Chi-squared or Fisher´s exact test was used to compare categorical variables and the Kruskal-Wallis test was used to compare continuous variables that were not normally distributed. The one-way ANOVA test was used for normally distributed, continuous variables. Univariable and multivariable logistic regression analysis were performed to identify predictors of in-hospital death. Variables that were statistically significant on univariable analysis were included in the multivariable model, with the exception of sex. Kaplan-Meier survival estimates and associated log-rank p-values were also determined. A p-value of less than 0.05 was considered statistically significant and 95% confidence intervals were used. Statistical analysis was performed using Stata IC version 16.1 (StataCorp LLC, Texas, USA).

## Results

**General characteristics of the study population:** a total of 448 patients were screened for acid-base disorders at the time of ICU admission. Seventy-three patients were excluded due to age less than 18 years (n=29), readmissions (n=8) and missing clinical records (n=36). There were 375 patients included in the study, of whom 75.6% (n=282) were found to have an acid-base disorder at the time of ICU admission. Regarding the demographic data, there was a significant difference in age between the groups, with patients in the respiratory alkalosis group being older. There were no differences regarding sex, medical or surgical patients, HIV status or tuberculosis between groups. However, most of the patients in each group had medical rather than surgical diagnoses (Table 1).

**Table 1:** baseline characteristics at the time of ICU admission

	Totals, n%	Metabolic acidosis	Metabolic alkalosis	Respiratory acidosis	Respiratory alkalosis	Mixed disorder	No disorder	p-value
Total, n%	375 (100)	35 (100)	25 (100)	23 (100)	16 (100)	183 (100)	93 (100)	-
**Demographic data**								
Age (years), median (IQR)	39 (30 to 52)	37 (28 to 55)	44 (31 to 54)	42 (34 to 56)	49 (33 to 63)	37 (29 to 50)	40 (30 to 50)	**<0.01**
Female	181 (48.3)	23 (65.7)	11 (44.0)	10 (43.4)	9 (56.2)	82 (45.0)	46 (49.5)	0.31
**Discipline, n%**								
Medical	261 (69.6)	21(60.0)	18 (72.0)	17 (73.9)	13 (81.2)	130 (71.4)	62 (66.7)	0.67 -
Surgical	114 (30.4)	14 (40.0)	7 (28.0)	6 (26.1)	3 (18.8)	52 (28.6)	31 (33.3)	
**Comordities, n%**								
Hypertension	92 (24.5)	9 (25.7)	3 (12.0)	8 (34.8)	7 (43.7)	39 (21.3)	26 (28.0)	0.14
Diabetes mellitus	55 (14.7)	5 (14.3)	3 (12.0)	5 (21.7)	3 (18.7)	27 (14.7)	12 (12.9)	0.88
CVD	5 (1.3)	0 (0)	0 (0)	1 (4.3)	1 (6.2)	0 (0)	3 (3.2)	**0.03**
Chronic lung disease	35 (9.3)	0 (0)	1 (4.0)	5 (21.7)	1 (6.2)	17 (9.3)	11 (11.8)	
CKD/ESKD	7 (1.9)	0 (0)	0 (0)	0 (0)	2 (12.5)	4 (2.2)	1 (1.1)	0.18
Chronic liver disease	1 (0.3)	0 (0)	0 (0)	0 (0)	0 (0)	1 (0.5)	0 (0)	1.00
Malignancy	10 (2.7)	1 (2.9)	0 (0)	1 (4.3)	1 (6.2)	5 (2.7)	2 (2.1)	0.68
HIV	91 (24.3)	9 (25.7)	8 (32.0)	4 (17.0)	3 (18.7)	52 (8.4)	15 (18.3)	0.27
TB	38 (10.1)	1 (2.9)	3 (12.0)	3 (13.0)	1 (6.3)	23 (12.6)	7 (8.4)	0.51
Other	86 (22.9)	7 (20.0)	8 (32.0)	6 (26.1)	2 (12.5)	36 (19.7)	27 (29.0)	0.36
**Clinical data**								
**Primary diagnosis**								
Pneumonia	81 (21.6)	1 (2.9)	4 (16.0)	8 (34.8)	6 (37.5)	43 (23.5)	19 (20.4)	**0.01**
Sepsis	59 (15.7)	11 (31.4)	2 (8.0)	2 (8.7)	1 (6.2)	33 (18.0)	10 (10.7)	0.05
Obstructive lung disease	16 (4.3)	0 (0)	1 (4.0)	3 (13.0)	0 (0)	9 (4.9)	3 (3.2)	0.28
Cardiac disease	25 (6.7)	4 (11.4)	0 (0)	4 (17.4)	1 (6.2)	11 (6.0)	5 (5.4)	0.16
Neurological disorder	24 (6.4)	1 (2.9)	5 (20.0)	0 (0)	2 (12.5)	8 (4.4)	8 (8.6)	**0.03**
Toxin ingestion/drug overdose	59 (15.7)	8 (22.9)	4 (16.0)	1 (4.3)	3 (18.7)	25 (13.7)	18 (19.3)	0.35
Trauma	48 (12.8)	2 (5.7)	5 (20.0)	2 (8.7)	1 (6.2)	18 (19.8)	20 (21.5)	0.05
Other	63 (16.8)	8 (22.9)	3 (16.0)	3 (13.0)	2 (12.5)	36 (19.7)	10 (10.7)	0.43
APACHE II score median (IQR)	20 (14 to 27)	22 (13 to 31)	21 (15 to 23)	21 (11 to 34)	20 (14.5 to 27)	21 (15 to 28)	19 (13 to 25)	**<0.01**
Mechanical ventilation	372 (99.2)	34 (97)	25 (100)	23 (100)	16 (100)	182 (99)	92 (98.9)	0.62
Systolic BP (mmHg), median (IQR)	124 (107 to 143)	114 (96 to 147)	119 (110 to 128)	124(112 to 146)	125.5 (118.5 to 156)	126 (107 to 144)	120 (110 to 140)	**<0.01**
Diastolic BP (mmHg), median (IQR)	70 (57 to 84)	64 (49 to 75)	65 (58 to 80)	74 (64 to 94)	78.5 (69 to 90)	71 (56 to 85)	70 (58 to 81)	**<0.01**
MAP	88 (75 to 104)	81 (67 to 98)	84 (78 to 97)	91 (84 to 111)	93.5 (83.5 to 112)	88 (74 to 106)	88 (75 to 102)	**<0.01**
AKI	178 (47.5)	25 (71.4)	10 (40.0)	14 (60.9)	7 (43.8)	88 (48.1)	34 (36.6)	**0.01**
Dialysis	27 (7.2)	3 (8.6)	2 (8.0)	0 (0)	1 (6.2)	18 (9.8)	3 (3.2)	0.27

Abbreviations: CVD, cardiovascular disease; CKD/ESKD, chronic kidney disease/end-stage kidney disease; HIV, human immunodeficiency virus; TB, tuberculosis; APACHE II, acute physiology and chronic health evaluation II score; BP, blood pressure; MAP, mean arterial pressure; AKI, acute kidney injury. Other comorbidities include epilepsy, gout, dyslipidaemia, autoimmune disease and psychiatric disorders

**Figure 1:**
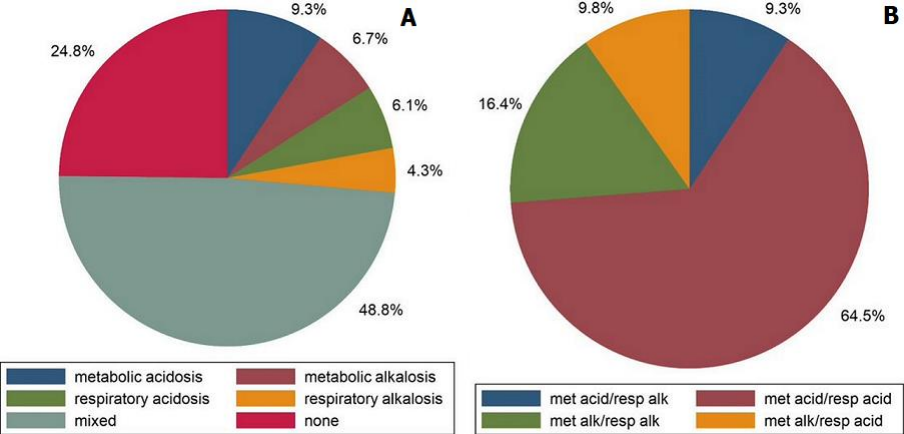
pie charts indicating the proportions of categories of acid-base disorders for the entire cohort (A) and for mixed disorders (B)

**Frequency and types of acid-base disorders:** mixed acid-base disorders were the most common at 48.8% (n=183) (Figure 1A). Within the mixed disorders, metabolic acidosis-respiratory acidosis was the most common (64.5%) (Figure 1B).

**Differences in clinical data between acid-base disorders:** regarding the clinical data, patients with a metabolic acidosis had higher APACHE II scores, lower systolic and diastolic blood pressures as well as lower mean arterial pressures and a greater proportion with acute kidney injury. There were no differences in the proportion receiving mechanical ventilation or dialysis. The most common primary diagnoses for ICU admission included pneumonia, sepsis other than pneumonia and toxin ingestion/drug overdose (Table 1). Pneumonia was more common in patients with respiratory acidosis (p = 0.01) while neurological disorders were more common in patients with metabolic alkalosis (p = 0.03).

**Differences in laboratory data between acid-base disorders:** regarding the laboratory data, there were significant differences between the groups for most of the arterial blood gas parameters; however, there was no difference in the partial pressure of oxygen between groups (Table 2). Patients with a metabolic acidosis had the highest median lactate concentration as well as a higher proportion of patients with a lactate concentration of ≥5 mmol/L (lactic acidosis). The AG/corrected AG could only be calculated in 78 patients with either a metabolic acidosis or mixed disorder (metabolic acidosis-respiratory acidosis/alkalosis) because the serum albumin concentrations were not measured in the remaining patients. Ninety-seven percent (97%) (n=76) of patients had a high corrected AG metabolic acidosis. There were no differences in the AG or corrected AG between the metabolic acidosis and mixed disorder groups. The metabolic acidosis group had a higher serum potassium concentration (4.6 (IQR 3.8 to 5.4) mmol/L) as well as higher serum urea (10.7 (IQR 5.8 to 25.0) mmol/L) and creatinine concentrations (162 (IQR 87 to 306) µmol/L).

**Table 2:** arterial blood gas and other laboratory data for patients grouped by acid-base disorder at ICU admission

	Metabolic acidosis	Metabolic alkalosis	Respiratory acidosis (Acute)	Respiratory alkalosis		Mixed disorder	No disorder	p-value
				Acute	Chronic			
Total, n%	35 (100)	25 (100)	23 (100)	5 (100)	11 (100)	183 (100)	93 (100)	-
pH	7.30 (7.26 to 7.33)	7.45 (7.43 to 7.45)	7.26 (7.21 to 7.29)	7.52 (7.48 to 7.52)	7.45 (7.44 to 7.46)	7.30 (7.21 to 7.37)	7.40 (7.38 to 7.41)	**<0.01**
Actual bicarbonate, mmol/L	15.8 (13.3 to 18.8)	30.5 (29.2 to 31.3)	26.6 (25.5 to 28.7)	22.4 (22.0 to 23.8)	20.2 (18.5 to 21.2)	24.1 (18.5 to 28.8)	24.8 (22.8 to 27.3)	**<0.01**
Standard bicarbonate, mmol/L	17.7 (14.7 to 20.2)	29.3 (28.7 to 30)	24.5 (23.6 to 25.3)	25.4 (24.7 to 25.5)	22.4 (21.2 to 22.9)	22.7 (18.6 to 27.7)	25.2 (23.0 to 26.8)	**<0.01**
PaCO_2_, kPa	4.1(3.6 to 4.7)	6.0(5.6 to 6.1)	7.6 (7.2 to 9.5)	4.0 (3.6 to 4.1)	3.9 (3.5 to 4.1)	6.1 (5.1 to 7.5)	5.3 (4.8 to 5.7)	**<0.01**
Standard base excess, mmol/L	-10.8 (13.6 to -6.9)	6.2 (5.2 to 7.3)	-0.2 (-1.2 to 1.2)	-0.9 (-1.0 to 0.3)	-3.8 (-5.3 to -3.1)	-3.2 (-8.5 to 3.8)	0.4 (-2.4 to 2.5)	**<0.01**
PaO_2_, kPa	13.7 (11.2 to 20.7)	12.3 (10.4 to 16.4)	10.9 (8.8 to 14.8)	16.9 (10.3 to 22.0)	11.2 (9.2 to 13.1)	12.3 (9.6 to 18.4)	13.0 (10.4 to 19.5)	0.39
SaO_2_, %	96.7 (94.5 to 99)	98 (96 to 99)	94 (90 to 96.9)	97.0 (96.0 to 99.0)	96.0 (95.0 to 98.0)	96 (92.1 to 99)	97 (95 to 99)	**0.02**
Lactate, mmol/L	2.7 (1.2 to 5.5)	1.4 (1.0 to 1.7)	1.8 (1.0 to 2.6)	1.0 (0.9 to 1.4)	1.8 (1.4 to 2.6)	1.8 (1.0 to 4.1)	1.5 (1.1 to 2.3)	**0.03**
Lactate categories, mmol/L								
0-2	15 (42.9)	20 (80.0)	13 (56.5)	5 (100)	7 (63.6)	101 (55.2)	64 (68.8)	**<0.01**
2.1-4.9	9 (25.7)	4 (16.0)	10 (43.5)	0 (0)	4 (36.4)	50 (27.3)	25 (26.9)	
≥ 5	11 (31.4)	1 (4.0)	0 (0)	0 (0)	0 (0)	32 (17.5)	4 (4.3)	
Albumin, g/L	26.5 (21 to 34)	31 (27 to 37)	33 (25 to 37)	37.5 (27.0 to 64.5)	26 (24.0 to 36.0)	27 (22 to 34)	30 (23.5 to 37)	0.31
AG, mmol/L, mean (SD)	24.9 (6.7)	-	-	-	-	24.6 (7.9)	-	0.85
Corrected AG, mmol/L, mean (SD)	28.3 (6.7)	-	-	-	-	28.3 (7.7)	-	0.98
Sodium, mmol/L	139 (137 to 143)	141 (138 to 146)	140 (137 to 143)	140 (138 to 141)	138 (137 to 145)	139 (135 to 143)	140 (137 to 143)	0.21
Potassium, mmol/L	4.6 (3.8 to 5.4)	3.9 (3.5 to 4.4)	4.4 (3.9 to 4.9)	3.9 (3.7 to 4.5)	4.2 (3.8 to 4.4)	4.4 (3.8 to 5.1)	4.2 (3.7 to 4.7)	**0.01**
Chloride, mmol/L	104 (100 to 109)	102 (99 to 104.5)	102 (98 to 105)	99.5 (97.5 to 104)	105 (105 to 110)	101 (96 to 105)	102.5 (98 to 107)	0.07
Urea, mmol/L	10.7 (5.8 to 25)	6.8 (4 to 12.2)	8.2 (4.7 to 12.9)	4.6 (4.5 to 9.5)	6.8 (3.4 to 18.1)	6.7 (4.5 to 14.4)	5.5 (3.8 to 10.9)	**0.02**
Creatinine, µmol/L	162 (87 to 306)	77 (62 to 133)	105 (52 to 131)	45 (45 to 112)	83 (56 to 202)	97 (64 to 233)	78 (58 to 119)	**<0.01**
Hb, g/dL, mean (SD)	11.1 (2.9)	11.3 (1.8)	11.5 (2.3)	11.0 (3.4)	12.0 (1.7)	11.2 (2.7)	11.6 (2.3)	0.86

Abbreviations: PaCO_2_, partial pressure of arterial carbon dioxide; PaO_2_, partial pressure of arterial oxygen; SaO_2_, arterial oxygen saturation; AG, anion gap; Hb, haemoglobin; numbers with ranges in parentheses represent median and interquartile ranges.

**Figure 2:**
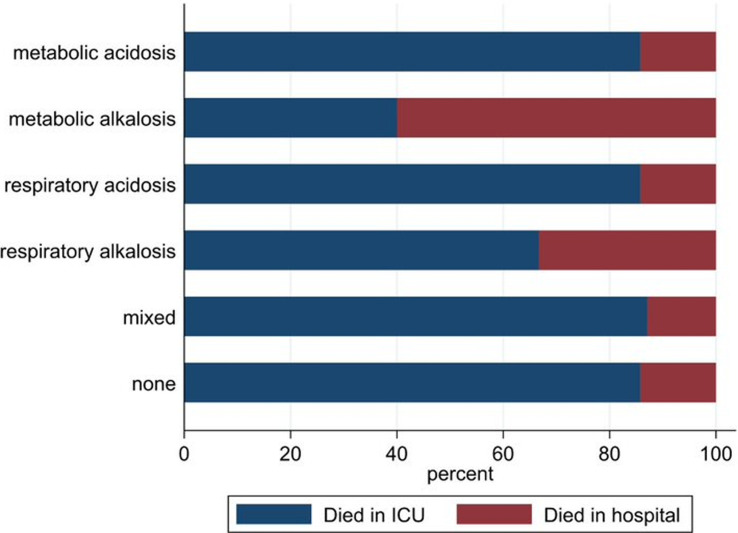
proportions of ICU and in-hospital deaths by acid-base disorders

**ICU and in-hospital death:** a total of 25.0% (n=94) patients died during hospitalisation. Most of the deaths occurred in the ICU (n=83). The median time to death in the ICU was 2 (IQR 1-5) days, while the median time to in-hospital death for patients surviving the ICU was 11 (IQR 8-23) days. There were no differences in ICU (p = 0.35) or in-hospital death (p = 0.32) by acid-base disorder (Figure 2).

**Figure 3:**
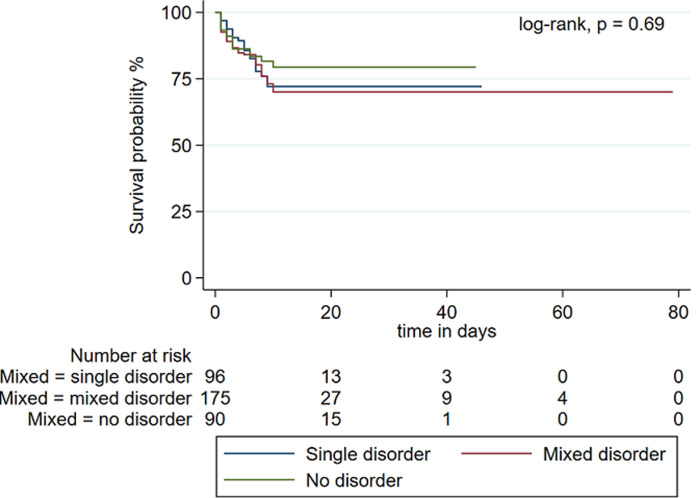
Kaplan-Meier survival analysis comparing patients with single, mixed and no acid-base disorder

**Predictors of in-hospital death:** on univariable logistic regression the following variables were statistically significant: age, APACHE II score, lactate concentration categories, AKI and the corrected AG. However, only male sex (aOR: 5.8, 95% CI 1.55-21.42; p < 0.01), APACHE II score (aOR: 1.17, 95% CI 1.06-1.30; p < 0.01) and the corrected AG (aOR: 1.14, 95% CI 1.02-1.27, p = 0.02) were predictors on multivariable analysis (Table 3). There were no differences in mortality on Kaplan-Meier survival estimates for patients with single, mixed or no acid-base disorder (log-rank, p = 0.69) (Figure 3). Variables associated with in-hospital death on Kaplan-Meier survival analyses included medical causes for ICU admission (log-rank, p < 0.01), AKI (log-rank, p < 0.01), metabolic acid-base disorders (log-rank, p = 0.01) and lactic acidosis (log-rank, p < 0.01).

**Table 3:** univariable and multivariable logistic regression for in-hospital death

Model	Univariable		Multivariable	
	OR (95% CI)	p-value	OR (95% CI)	p-value
Age	1.02 (1.00-1.03)	**0.01**	0.98 (0.93-1.02)	0.33
Male sex	1.2 (0.76-1.94)	0.42	5.8 (1.55-21.42)	**< 0.01**
HIV positive	1.2 (0.69-2.06)	0.52		
Tuberculosis	1.8 (0.88-3.72)	0.11		
APACHE II score	1.12 (1.09-1.15)	**< 0.01**	1.17 (1.06-1.30)	**< 0.01**
Type of acid-base disorder (reference: metabolic acidosis)				
Metabolic alkalosis	0.8 (0.20-2.95)	0.69		
Respiratory acidosis	1.7 (0.52-5.90)	0.37		
Respiratory alkalosis	2.4 (0.64-8.87)	0.19		
Mixed	1.5 (0.63-3.76)	0.34		
None	1.03 (0.39-2.71)	0.96		
Lactate category (reference: 0-2 mmol/L)				
2.1-4.9 mmol/L	2.3 (1.37-4.05)	**< 0.01**	1.2 (0.22-6.34)	0.84
≥ 5.0 mmol/L	4.5 (2.33-8.80)	**< 0.01**	1.3 (0.25-6.62)	0.76
AKI - Yes	3.5 (2.15-5.88)	**< 0.01**	2.7 (0.21-36.5)	0.44
Dialysis - Yes	1.8 (0.81-4.19)	0.14		
Corrected AG	1.15 (1.06-1.25)	**< 0.01**	1.14 (1.02-1.27)	**0.02**

Abbreviations: OR, odds ratio; 95% CI, 95% confidence interval; HIV, human immunodeficiency virus; APACHE II, acute physiology and chronic health evaluation II score; AKI, acute kidney injury

## Discussion

In this study, our primary aims were to determine the frequency and types of acid-base disorders at the time of admission to the ICU and to assess whether acid-base disorders were associated with ICU and in-hospital mortality. We found that mixed acid-base disorders were the most frequent disorder at the time of admission to the ICU; however, we did not find any association between the acid-base disorder at admission to the ICU and in-hospital death.

Our patients were young and had few comorbid diseases, reflecting the experience of other low-to-middle income countries (LMICs). A study that assessed the worldwide burden of critical illness found that LMICs tended to have lower rates of comorbid diseases apart from diabetes mellitus [18]. The mortality rate for patients from the African continent was only 20.7% and overall, LMICs had lower ICU and in-hospital mortality rates when compared with upper-middle-income countries. Other than having fewer comorbidities, patients from Africa were younger, had lower APACHE II scores, and less than half received mechanical ventilation. The young age of our patients is probably the result of selection bias because of limited resources. A multivariable regression model identified the corrected AG as a predictor of in-hospital death. Since this high corrected AG remained a predictor even after adjusting for lactic acidosis, other causes for an added acid type of metabolic acidosis contributed to this risk. These may have included AKI, toxin ingestion such as ethylene glycol poisoning, and ketoacidosis. Similarly, another study found that patients with a high corrected AG metabolic acidosis at the time of admission had a higher mortality relative to those patients without any acid-base disorder [19]. Therefore, although it is the primary diagnosis that predominantly determines the clinical outcome, the presence of a high AG metabolic acidosis is a strong predictor of an adverse outcome.

Mixed acid-base disorders were the most common acid-base disorder at the time of ICU admission. The reason for this finding is probably multifactorial. Our hospital provides tertiary services for half of the Western Cape province of South Africa. Many patients initially access healthcare services at the primary or secondary level, and critically ill patients may be managed in our medical emergency unit and/or high care ward before being referred to the ICU. Treatments administered to patients during their initial stages of hospitalisation may have influenced the primary acid-base disorder. Since many patients were initially managed outside of the ICU, only patients whose condition worsened and were considered candidates for ICU admission, were referred to intensive care services. These patients were more likely to have developed a second acid-base disorder due to the progression of the underlying condition. Mixed metabolic acidosis-respiratory acidosis constituted two-thirds of mixed disorders. A quarter of the patients with mixed metabolic acidosis-respiratory acidosis were mechanically ventilated due to pneumonia. Since pneumonia may progress to acute respiratory distress syndrome, lung-protective ventilation is often used. This involves the use of low tidal volumes to reduce ventilator-associated lung injury. As a result, “permissive hypercapnia” is allowed, with subsequent respiratory acidosis [20].

The second most common acid-base disorder was metabolic acidosis. These patients were the most critically ill with higher APACHE II scores, lower blood pressures, and the highest proportion of patients with AKI and lactic acidosis. The reason for these findings was probably linked to the primary diagnosis. Sepsis and toxin ingestion/drug overdose constituted more than half of the primary diagnoses. These are frequently complicated by AKI and lactic acidosis. Also, since these patients were haemodynamically unstable, vasopressor support such as intravenous adrenaline was another factor that may have contributed to their metabolic acidosis through the stimulation of lactate production. None of the patients had chronic respiratory acidosis. Chronic lung diseases frequently encountered in our emergency department include chronic obstructive pulmonary disease and post-tuberculous structural lung disease. Many of these patients requiring intensive care present with advanced, end-stage disease with limited access to lung transplantation [21]. In view of the limited critical care resources available, these patients are frequently not offered intensive care. In the current study, only five patients with an acute exacerbation of hronic obstructive pulmonary disease (COPD) and two patients with haemoptysis secondary to post-tuberculous structural lung disease were identified. The remainder were admitted for severe asthma exacerbations. These study had a few strengths and limitations. This was a large, single centre study performed at a tertiary hospital. Due to the retrospective study design, missing data may have introduced bias. As a result of limited ICU resources, the decision to admit patients was guided by local policies. This may have influenced the patient profile as those deemed to have a poor prognosis were not admitted to the ICU. Also, our cohort of patients was young with few comorbid diseases; therefore, our results and outcomes may not be generalisable.

## Conclusion

We found no association between acid-base disorders at the time of ICU admission and ICU or in-hospital death; therefore, in our setting, acid-base disorders at the time of ICU admission should not be used to predict the outcome of patients requiring intensive care. Mixed acid-base disorders were the most common, with metabolic acidosis-respiratory acidosis being the most common type of mixed disorder. The corrected AG was a predictor of death after adjusting for lactic acidosis, suggesting that other causes of a high AG metabolic acidosis are important in determining prognosis.

### What is known about this topic

- *Acid-base disorders are very common in critically ill patients;*
- *There is no single acid-base disorder that has consistently been shown to be the most common in the intensive care unit (ICU);*
- *A small study reported that 70% of patients had a mixed acid-base disorder at ICU admission*.

### What this study adds

- *We found that 75.6% of patients had an acid-base disorder at the time of ICU admission;*
- *Mixed acid-base disorders (48.8%) were the most common with metabolic acidosis-respiratory acidosis being the common mixed disorder;*
- *There was no association between acid-base disorders at the time of ICU admission and ICU or in-hospital death*.
